# Combined gemcitabine and S-1 chemotherapy for treating unresectable hilar cholangiocarcinoma: a randomized open-label clinical trial

**DOI:** 10.18632/oncotarget.8590

**Published:** 2016-04-05

**Authors:** Hao Li, Zheng-Yun Zhang, Zun-Qiang Zhou, Jiao Guan, Da-Nian Tong, Guang-Wen Zhou

**Affiliations:** ^1^ Department of Surgery, Shanghai Jiao Tong University Affiliated First People's Hospital, Shanghai, 200080, China; ^2^ Department of Surgery, Shanghai Jiao Tong University Affiliated Sixth People's Hospital, Shanghai, 200233, China

**Keywords:** gemcitabine, S-1, hilar cholangiocarcinoma, chemotherapy, CA19-9

## Abstract

Although the combination of cisplatin and gemcitabine (GEM) is considered the standard first-line chemotherapy against unresectable hilar cholangiocarcinoma (HC), its efficacy is discouraging. The present randomized open-label clinical trial aimed to evaluate the efficacy and safety of the GEM plus S-1 (GEM-S-1) combination against unresectable HC. Twenty-five patients per group were randomly assigned to receive GEM, S-1 or GEM-S-1. Neutropenia (56%) and leukopenia (40%) were the most common chemotherapy-related toxicities in the GEM-S-1 group. Median overall survival (OS) in the GEM-S-1, GEM and S-1 groups was 11, 10 and 6 months, respectively. GEM plus S-1 significantly improved OS compared to S-1 monotherapy (OR=0.68; 95%CI, 0.50–0.90; *P*=0.008). Median progression-free survival (PFS) times in the GEM-S-1, GEM and S-1 groups were 4.90, 3.70 and 1.60 months, respectively. GEM plus S-1 significantly improved PFS compared to S-1 monotherapy (OR=0.50; 95%CI, 0.27–0.91; *P*=0.024). Response rates were 36%, 24% and 8% in the GEM-S-1, GEM and S-1 groups, respectively. A statistically significant difference was found in response rates between the gemcitabine-S-1 and S-1 groups (36% vs 8%, *P*=0.017). Patients with CA19-9<466 U/ml were more responsive to chemotherapeutic agents than those with CA19-9≥571 U/ml (88.9% vs 0%, *P*<0.001). We conclude that the combination of GEM plus S-1 provides a better OS, PFS and response rate than S-1 monotherapy, but it did not significantly differ from GEM monotherapy. (ChiCTR-TRC-14004733).

## INTRODUCTION

Hilar cholangiocarcinoma (HC) is the most frequently identified cholangiocarcinoma and accounts for 58–66% of cholangiocarcinoma cases in China [1, 2]. Gemcitabine (GEM) and fluorouracil (as single agents or in combination) were recommended by the National Comprehensive Cancer Network (NCCN) for the treatment of advanced cholangiocarcinoma [3]. In fact, GEM-based combined chemotherapy has been demonstrated to improve survival in resectable HC [4]. More recently, cisplatin plus GEM resulted in a significant survival advantage without substantial toxicity compared with GEM alone in patients with locally advanced or metastatic cholangiocarcinoma, gallbladder cancer or ampullary cancer [5]. Thus, cisplatin and GEM in combination is now considered the standard first-line chemotherapy [6]. However, the efficacy of this treatment regimen against unresectable HC is discouraging. The combination of GEM and S-1 has been widely used in patients with pancreatic cancer [7-9], but outcomes for this treatment in cholangiocarcinoma are still unknown. S-1 is a fourth generation oral fluoropyrimidine prodrug that includes tegafur, 5-chloro-2,4-dihydropyrimidine (CDHP; a dihydropyrimidine dehydrogenase inhibitor) and potassium oxonate [10]. S-1 has a favourable toxicity profile and can be safely used in cholangiocarcinoma patients with hyperbilirubinemia [11]. S-1 monotherapy was reportedly effective in patients with advanced biliary tract cancer [12-14]. A preclinical study indicated that S-1 and GEM had synergistic effects [15], and a multicenter phase II study showed that this combination was promising for treating advanced biliary tract cancer [16]. However, it had a relative small sample size (a total of 35 patients) and among the 35 patients, 14 patients had gallbladder cancer, 14 had intrahepatic cholangiocarcinoma and 7 patients had received previous surgical resection. Moreover, it did not compare the gemcitabine and S-1 combination therapy with gemcitabine monotherapy, or S-1 monotherapy. Another study showed that GEM and S-1 had good efficacy against resected biliary carcinoma, it could be an adjuvant chemotherapy to surgical resection of advanced biliary carcinoma [17]. For unresectable HC, the combination of GEM and S-1 had not been studied. The present prospective study assessed 75 cases of unresectable HC to evaluate the efficacy and safety of GEM and S-1 combined chemotherapy.

## RESULTS

### Patients

**Figure 1:**
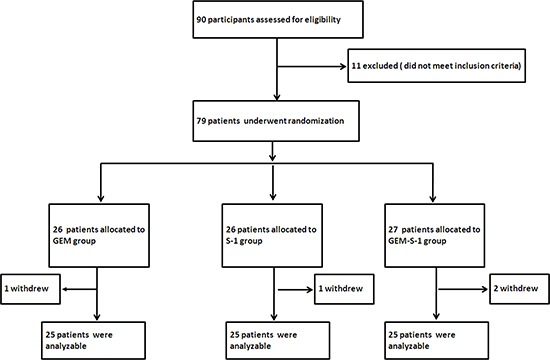
Patient selection flow chart Seventy-five out of 79 patients completed the analysis, including 25 each in the GEM, S-1 and GEM-S-1 groups. GEM, gemcitabine; GEM-S-1, gemcitabine plus S-1.

A total of 90 patients with consecutive unresectable HC were treated by our surgical team. Eleven of these patients did not meet the inclusion criteria: 7 had prior history of chemotherapy or radiotherapy; 3 presented with active infection and 1 with severe drug hypersensitivity. The remaining 79 patients were randomly allocated to the study groups. However, four patients discontinued the treatment, because of financial hardship or the unwillingness to continue the treatment because of advanced disease. Seventy-five out of 79 patients (94.9%) completed the analysis, with 25 subjects in each treatment group (GEM, S-1, and GEM-S-1) (Figure 1).

Patients' baseline demographic and clinical characteristics were summarized in Supplementary Table S1. No significant differences were found among the three groups in age, gender, carbohydrate antigen 19-9 (CA19-9), carcinoembryonic antigen (CEA), Bismuth-Corlette classification, UICC stage, metastases or biliary drainage (*P*>0.05).

### Patient survival

**Figure 2:**
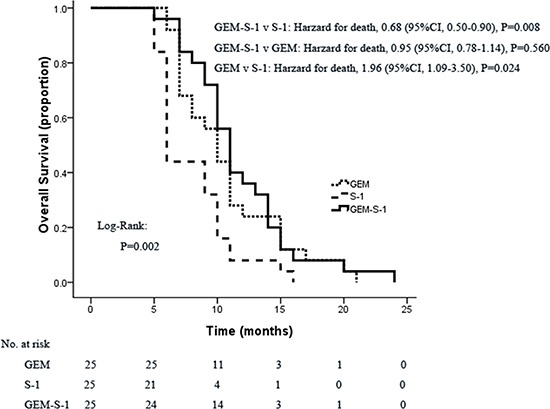
Kaplan-Meier curves of OS according to treatment group GEM, gemcitabine; GEM-S-1, gemcitabine plus S-1; 95%CI, 95% confidence interval.

**Figure 3:**
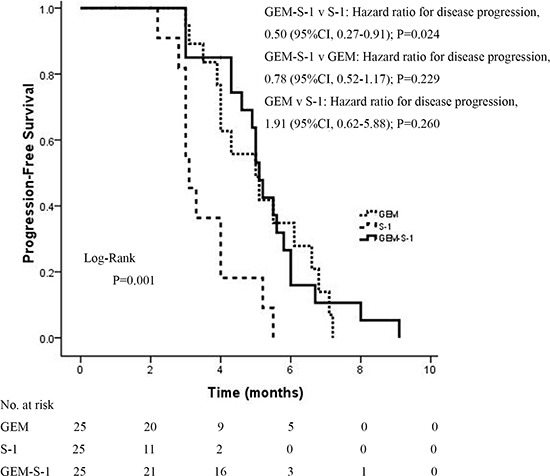
Kaplan-Meier curves of PFS according to treatment group GEM, gemcitabine; GEM-S-1, gemcitabine plus S-1; 95%CI, 95% confidence interval.

One-year survival rates for the GEM-S-1, GEM and S-1 groups were 40%, 28% and 8%, respectively (*P*=0.032) (Table 1). Significant differences were found between the GEM-S-1 and S-1 groups (40% vs. 8%, *P*=0.008), but not the GEM-S-1 and GEM (40% vs. 28%, *P*=0.370). Although one-year survival rate was threefold higher in the GEM group compared to S-1 treated patients (28% vs. 8%), the difference did not reach statistical significance (*P*=0.066). Median OS was 11.0 months (95%CI, 9.80–12.20 months) in the GEM-S-1 group, 10 months (95%CI, 8.38–11.62 months) in the GEM group, and 6 months (95%CI, 5.51–6.49 months) in the S-1 group (Figure 2). These data indicated that GEM plus S-1 significantly improved OS compared with S-1 monotherapy (hazard ratio for death, 0.68; 95%CI, 0.50–0.90; *P*=0.008). GEM monotherapy also significantly improved OS compared with S-1 monotherapy (hazard ratio for death, 1.96; 95%CI, 1.09-3.50; *P*=0.024). However, GEM plus S-1 did not improve OS at a statistically significant level compared with GEM monotherapy (hazard ratio for death, 0.95; 95% CI, 0.78-1.14; *P*=0.560) (Figure 2). Median PFS were 4.90 months (95%CI, 1.30–8.77 months), 3.70 months (95%CI, 1.03–7.17 months), and 1.60 months (95%CI, 0.66–5.41 months) in the GEM-S-1, GEM and S-1 groups, respectively, indicating significant differences among the three groups (*P*=0.001). GEM plus S-1 significantly improved PFS compared with S-1 monotherapy (hazard ratio for disease progression, 0.50; 95%CI, 0.27-0.91; *P*=0.024). However, GEM plus S-1 did not improve PFS at a statistically significant level compared with GEM monotherapy (hazard ratio for death, 0.78; 95% CI, 0.52-1.17; *P*=0.229). Also, GEM monotherapy did not improve PFS compared with S-1 monotherapy (hazard ratio for death, 1.91; 95%CI, 0.62-5.88; *P*=0.260) (Figure 3).

**Table 1:** Chemotherapeutic efficacies

Variable	Total(n=75)	GEM(n=25)	S-1(n=25)	GEM-S-1(n=25)	P			
	No (%)	No (%)	No (%)	No (%)	Total P	GEM-S-1 vs GEM	GEM-S-1 vs S-1	GEM vs S-1
CR	4(5.3%)	2(8.0%)	0	2(8.0%)	0.785	1.000	0.490	0.490
PR	13(17.3%)	4(16.0%)	2(8.0%)	7(28.0%)	0.169	0.306	0.138	0.667
SD	28(37.3%)	9(36.0%)	9(36.0%)	10(40.0%)	0.945	0.771	0.771	1.000
Prolonged SD (≥6 months)	7 (9.3%)	2(8.0%)	1(4.0%)	4(16.0%)	0.332	0.667	0.349	1.000
PD	30(40.0%)	10(40.0%)	14(56.0%)	6(24.0%)	0.069	0.225	0.021	0.258
RR	22.7%	24.0%	8.0%	36.0%	0.060	0.355	0.017	0.247
DCR	32.0%	32.0%	12.0%	52.0%	0.010	0.152	0.002	0.088
One-year survival rate	25.3%	28.0%	8.0%	40.0%	0.032	0.370	0.008	0.066

Abbreviations: GEM, gemcitabine; GEM-S-1, gemcitabine plus S-1; CR: Complete response; PR: Partial response; SD: Stable disease; PD: Progressive disease; RR: Response rate; DCR: Disease control rate; DCR=CR+PR+Prolonged SD.

### Response to therapy

As shown in Table 1, the RR was 36% in the GEM-S-1 group, 24% in GEM treated patients, and 8% in the S-1 group. The RR between the GEM-S-1 and S-1 groups was significantly different (*P*=0.017), but not between the GEM and GEM-S-1 (*P*=0.355) or GEM and S-1 groups (*P*=0.247). There were four patients with complete response (CR) (5.3%), including two patients in GEM-S-1 group and two patients in GEM group. CR duration ranged from 15–18 months in the GEM group and 18–22 months in the GEM-S-1 group. In the GEM-S-1 group, 7 patients (28%) experienced partial response (PR) with response durations of 10–14 months, 4 patients in GEM group had response durations of 12–15 months, and 2 patients in S-1 group had response durations of 13–14 months. The incidence of stable disease (SD) was similar in each group (*P*=0.945), and included nine patients (36%) in the GEM and S-1 groups, and ten patients (40%) in the GEM-S-1 group. Seven patients experienced prolonged stable disease (≥6 months), including two in the GEM group, one in the S-1 group, and four in the GEM-S-1 group. No significant differences were observed between any two groups.

### Univariate and multivariate analysis of one-year survival

Clinicopathological factors and chemotherapy regimens were investigated to determine whether they were of prognostic significance. CA19-9 level (*P*<0.001), UICC Stage (*P*<0.001), liver metastases (*P*=0.003), and chemotherapy regimens (P=0.032) were associated with patients' one-year survival (Supplementary Table S2). These factors were entered into multivariate analysis; UICC Stage (*P*=0.040) and CA19-9 (*P*<0.001) remained independently associated with one-year survival. The risk of death within one year in stage IV patients was 3.831 times higher than that in patients in stage III according to UICC stage, and the risk of death within one year in patients with CA19-9 level above 500 U/ml was 55.556 times higher than that of patients with CA19-9 level below 500 U/ml (Supplementary Table S2).

### Influencing factors influencing chemotherapeutic effect

Univariate analysis showed that UICC stage (III), CA19-9 and liver metastases influenced the effects of chemotherapy (*P*<0.001) (Table 2). Patients responsive to chemotherapy had lower CA19-9 levels than unresponsive individuals (382 U/ml vs 614 U/ml, *P*<0.001). After multivariate analysis, CA19-9 remained independently associated with chemotherapeutic effects of (*P*=0.025) (Table 2).

**Table 2:** Univariate and multivariate analysis of indicators influencing chemotherapeutic effects

	Univariate analysis			Multivariate analysis	
	RR(n=17)	Non RR(n=58)	P	OR(95%CI)	P
Age	57.47±6.62	55.93±8.17	0.479		
Gender (Man)	11(64.7%)	43(74.1%)	0.541		
UICC stage (III)	14 (82.4%)	8(13.8%)	<0.001		
Bithmuth-Corlette classification(IV)	5(29.4%)	22(37.9%)	0.813		
CEA	2.49±0.89	2.49±0.76	0.982		
CA19-9	382.47±56.60	614.26±96.50	<0.001	0.901 (0.822-0.987)	0.025
Liver metastases	0	25 (43.1%)	<0.001		
Biliary drainage (ERCP)	10 (58.8%)	33 (56.9%)	0.888		
Chemotherapy regimens (GEM-S-1)	9 (52.9%)	16 (27.6%)	0.060		

Abbreviations: RR, response rate; UICC, International Union for Cancer Control; CEA, carcinoembryonic antigen; CA19-9, carbohydrate antigen 19-9; ERCP, endoscopic retrograde cholangiopancreatography; GEM-S-1, gemcitabine plus S-1; OR, odds ratio; CI, confidence intervals

When stratified by CA19-9 levels, 88.9% of patients with CA19-9<466 U/ml were responsive to chemotherapy. No patients with CA19-9 levels >571 U/ml were responsive to any of the chemotherapy regimens. Only 5.3% of patients with CA19-9 levels 466–571 U/ml were responsive to chemotherapy. RR values were significantly different among the three groups when stratified by CA19-9 level (*P*<0.001) (Table 3).

**Table 3:** Response rates stratified by carbohydrate antigen 19-9 level

	RR	P
CA19-9<466	88.9%(16-18)	
466≦CA19-9<571	5.3%(1-19)	<0.001
571≦CA19-9	0% (0-38)	

Abbreviations: RR, response rate; CA19-9, carbohydrate antigen 19-9

### Toxicity and side effects

Neutropenia (34.7%) and nausea (34.7%) were the most common toxic effects of chemotherapy (Table 4). Neutropenia was more common in the GEM-S-1 group compared with S-1 treated patients (56% vs. 8%, *P*<0.001), and in the GEM group compared with the S-1 group (40% vs. 8%, *P*=0.008). Similarly, leukopenia incidence was higher in the GEM-S-1 group in comparison with S-1 treated patients (40% vs. 4%, *P*=0.002); no significant differences were found between the GEM and S-1 groups (*P*=0.189). No significant differences in neutropenia or leukopenia were found between the GEM-S-1 and GEM groups (*P*>0.05). Liver function parameters, including alanine transaminase (ALT) and aspartate transaminase (AST) levels, were similar in the three groups (*P*=0.321). Elevated total bilirubin (hyperbilirubinemia) was found in 20% of patients treated with GEM-S-1 and in 12% of patients in each of the GEM and S-1 groups, with no significant differences (*P*=0.386). Thrombocytopenia rates were 20%, 12% and 4% in GEM-S-1, GEM and S-1 groups, respectively, again with no significant differences (*P*=0.258). Other side effects, including febrile neutropenia, low hemoglobin, and constitutional effects such as fatigue, rash, and anorexia, were the same among the three groups (*P*>0.05). Of the 75 patients, no one discontinued treatment because of toxicity. Side effects were, in most cases, transient and easily managed, and treatment was well tolerated. No patients died of treatment-related causes during the study. A total of five patients had dose reductions, two in the GEM group and three in the GEM-S-1 group. As appropriate, GEM dosage was reduced to 800 mg/m^2^ and that of S-1 was reduced to 20 mg/day in the subsequent cycles.

**Table 4:** Therapy-related toxicities and side effects

Toxicity and side effect	Grade 1-2 n (%)							
	Total(n=75) No (%)	GEM(n=25) No (%)	S-1(n=25) No (%)	GEM-S-1(n=25) No (%)	P			
					Total P	GEM-S-1 vs GEM	GEM-S-1 vs S-1	GEM vs S-1
**Constitutional**								
Fatigue	6(8.0%)	3(12.0%)	2(8.0%)	1(4.0%)	0.309	0.609	1.000	1.000
Rash	3(4.0%)	1(4.0%)	0	2(8.0%)	0.348	1.000	0.490	1.000
Anorexia	7(9.3%)	2(8.0%)	2(8.0%)	3(12.0%)	0.598	1.000	1.000	1.000
Diarrhea	3(4.0%)	1(4.0%)	1(4.0%)	1(4.0%)	1.000	1.000	1.000	1.000
Nausea	26(34.7%)	8(32.0%)	8(32.0%)	10(40.0%)	0.790	0.556	0.556	1.000
Vomiting	14(18.7%)	5(20.0%)	3(12.0%)	6(24.0%)	0.582	0.733	0.463	0.702
Mucositis-stomatitis	2(2.7%)	0	1(4.0%)	1(4.0%)	0.447	1.000	1.000	1.000
**Gastrointestinal-hepatology**								
Elevated ALT	10(13.3%)	3(12.0%)	2(8.0%)	5(20.0%)	0.321	0.702	0.417	1.000
Elevated AST	10(13.3%)	3(12.0%)	2(8.0%)	5(20.0%)	0.321	0.702	0.417	1.000
Hyperbilirubinaemia	11(14.7%)	3(12.0%)	3(12.0%)	5(20.0%)	0.386	0.702	0.702	1.000
**Haematologic**								
Leukopenia	16(21.3%)	5(20.0%)	1(4.0%)	10(40.0%)	0.008	0.123	0.002	0.189
Neutropenia	26(34.7%)	10(40.0%)	2(8.0%)	14(56.0%)	0.001	0.258	<0.001	0.008
Febrile neutropenia	3(4.0%)	2(8.0%)	0	1(4.0%)	0.639	1.000	1.000	0.490
Thrombocytopenia	9(12.0%)	3(12.0%)	1(4.0%)	5(20.0%)	0.258	0.702	0.189	0.609
Low hemoglobin	11(14.7%)	3(12.0%)	3(12.0%)	5(20.0%)	0.386	0.702	0.702	1.000

Abbreviations: GEM, gemcitabine; GEM-S-1, gemcitabine plus S-1; No, number; ALT, alanine aminotransferase; AST, aspartate aminotransferas

## DISCUSSION

Chemotherapy is the most effective approach to prolong survival (generally extended to 6–12 months) in patients with unresectable HC [18, 19]. Although the GEM plus S-1 combination clearly provides benefits in unresectable pancreatic cancer [20], discrepant findings have been published for this combination in unresectable HC [21, 22]. Therefore, the present study aimed to further assess the value of GEM plus S-1 in a comparatively large cohort of patients with unresectable HC.

Compared to S-1 monotherapy, GEM-S-1 therapy demonstrated significant and promising efficacy in terms of RR, median OS and PFS. The beneficial effects of S-1 monotherapy described by Suzuki, *et al.* [23] were not observed in our study. It should be noted that these authors assessed only one case. In accordance with our findings, S-1 monotherapy has not been advocated as first-line chemotherapy in the treatment of unresectable HC. As shown above, no significant differences in RR, median OS or PFS were found between the GEM-S-1 and GEM groups, although the GEM-S-1 combination showed slightly higher RR values (36% vs 24%). A similar pattern was observed for median OS (11 vs 10 months) and PFS (4.90 vs 3.70 months). These results are consistent with Sasaki, *et al.*, who reported that the median PFS and OS of GEM-S-1 and GEM treatments were nearly the same (5.6 vs. 4.3 months, and 8.9 vs. 9.2 months, respectively) [22]. However, GEM monotherapy significantly improved OS compared with S-1 treatment. Therefore, GEM is superior to S-1 as a first-line chemotherapeutic in the treatment of unresectable HC. The efficacy of GEM-S-1 observed here is similar to values reported for patients with pancreatic cancer, in which RR ranged between 21.6% and 32.4%, and median OS between 8.4 and 13.7 months [20, 24-27]. The probable mechanism by which GEM-S-1 increases the survival of patients with unresectable HC might lie in S-1, which has been shown to upregulate nucleoside transporter proteins that allow higher GEM intake into tumor cells [28]. However, the improvement did not appear to be significant, since GEM-S-1 and GEM monotherapy groups showed comparable outcomes.

In addition, neutropenia and leukopenia occurred more frequently in the GEM-S-1 group, in agreement with previous reports [22, 25]. In the present study, neutropenia was also more common in the GEM group compared with S-1 treated individuals. Myelosuppression, especially neutropenia, seems to be a major toxicity associated with GEM-based therapy. These side effects were temporary and could be alleviated by symptomatic treatment or dose reduction. Indeed, no patient died due to chemotherapy's side effects. Additionally, 20% of patients in the GEM-S-1 group experienced thrombocytopenia, slightly fewer than the 26% reported by Sudo, *et al.* in patients with unresectable pancreatic cancer [25]. For patients with severe thrombocytopenia, clinicians should be alert to the risk of bleeding and the dose of chemotherapy drugs reduced accordingly.

Multivariate analysis showed that CA19-9 levels can predict HC patient responsiveness to chemotherapy: higher CA19-9 levels correlated with reduced chemotherapy response, in agreement with other studies [29]. Patients were more responsive to chemotherapy when premedication CA19-9 levels were below 466 U/ml. At premedication CA19-9 levels higher than 571 U/ml, patients were unresponsive to any of the three treatment regimens.

Our study has several limitations. First, GEM and S-1 pharmacokinetics and pharmacodynamics may be different between Western and Eastern patients, which may reduce the generalizability of these findings. In addition, although the sample size used here was reasonably large due to HC rarity, these findings must be further evaluated in larger groups, which could be achieved in a multicenter trial. Most importantly, at the time this prospective study was launched, GEM and/or fluorouracil were recommended by the NCCN for the treatment of advanced cholangiocarcinoma, and the current regimen of GEM and cisplatin was not in use; therefore, we failed to include this important control in our studies. Despite limitations, this study demonstrated that administration of GEM-S-1 improves response rate, OS and PFS in patients with unresectable HC.

## MATERIALS AND METHODS

### Trial design and patients

This was a prospective, single-center, randomized, open-label clinical study of patients with unresectable HC. Eligible patients were randomly assigned to the GEM-S-1, GEM and S-1 groups via a computer-generated randomization list (Supplementary methods). The protocol was approved by the institutional review board of Shanghai Jiao Tong University. All patients provided signed informed consent before treatment. The study, which was conducted according to the principles of the Declaration of Helsinki, was registered in the Chinese Clinical Trial Registry (ChiCTR-TRC-14004733).

We assumed that within the two-year follow-up period, the one-year survival rate would be 30% in the GEM-S-1 group and 10% in the other two treatment groups (S-1 or GEM monotherapy). 18 patients per group (54 total) were needed for 90% power with a two-sided significance level of 0.05. Assuming a 40% drop-out rate, 90 patients were enrolled. From February 2009 to November 2012, patients with unresectable HC, as diagnosed by clinical pathological examination or typical radiographic findings at our hospital, were treated by our surgical team. The patient randomization process followed baseline testing, which included physical examination, blood tests, electrocardiogram and abdominal ultrasound. Eligible patients were randomly assigned to the GEM/S-1, GEM or S-1 groups in a 1:1:1 ratio by use of a computer-generated randomization list. To conceal individual patient group assignments, a trial/data manager who was not involved in patient recruitment generated the patient random allocation sequence via computer. GEM and S-1 were obtained from Lilly France Company (Fegersheim, France), and Taiho Pharmaceutical Co. Ltd (Tokyo, Japan), respectively.

### Patient enrollment and exclusion criteria

We enrolled patients from the Department of Surgery, Shanghai Jiao Tong University Affiliated Sixth People's Hospital, a tertiary-care institute. Patients were eligible if they met the following criteria: (1) HC diagnosed by clinical pathological examination or typical radiographic findings; (2) unresectable locally advanced or metastatic disease; (3) except for biliary drainage, no prior treatment for HC, including surgery, radiation or chemotherapy; (4) age>18 years; (5) capable of oral intake; (6) hematologic and biochemical parameters considered as follows: white blood count>3000/mm^3^, hemoglobin >9.0g/dL, platelet count >100000/mm^3^, total bilirubin<3.0 times the upper limit of normal (ULN), aspartate/alanine transaminases<5 times the ULN, and creatinine level<1.5 times the ULN. Exclusion criteria were as follows: (1) severe complications, such as active infection, cardiac or renal disease, marked pleural effusion, or ascites; (2) active gastrointestinal bleeding; (3) severe drug hypersensitivity; (4) active concomitant malignancy; (5) a prior history of chemotherapy or radiotherapy. Patients who discontinued treatment or could not attend scheduled evaluations were excluded.

### Treatment

All patients were treated within a four-week cycle. GEM group individuals were administered 1000 mg/m^2^ GEM intravenously over 30 min on days 1, 8 and 15. Patients in the S-1 group were administered medication orally twice daily for 14 days, followed by a 14-day rest during each four-week cycle. Three doses of S-1 were used based on body surface area (BSA): BSA≤1.25 m^2^, 80 mg per day; 1.25 m^2^>BSA<1.5 m^2^, 100 mg per day; BSA≥1.5 m^2^, 120 mg per day. Patients randomly allocated to the GEM-S-1 group received 1000 mg/m^2^ GEM intravenously over 30 min on days 1 and 15 and S-1 orally twice daily for two weeks followed by a two-week rest during each four-week cycle. Treatments were carried out until disease progression (via either clinical or radiologic evidence), unacceptable toxicity or patient withdrawal occurred. In the case of predefined toxicity, assessment of adverse events and dose adjustments were permitted.

### Adverse events and dose adjustments

Adverse effects were graded according to the National Cancer Institute Common Terminology Criteria for Adverse Events (NCI-CTCAE) version 4.02. GEM and S-1 were administered and dose-adapted according to the observed toxic effects. Treatment was continued until occurrence of unacceptable toxicity. GEM dosage was reduced to 800 mg/m^2^, and that of S-1 by 20 mg/day in the subsequent cycle, when patients experienced drug toxicity events such as grade 4 leukopenia or neutropenia, grade 4 or 3 thrombocytopenia or ≥grade 3 non-hematological toxicity not tolerated by the individual. If a patient required more than two dose reductions, treatment was discontinued.

### Response and assessments

Tumor responses were measured by spiral computed tomography or magnetic resonance imaging, performed at baseline and every two cycles (8 weeks) thereafter. Two radiologists and an adjudicator, none of whom were involved in this trial, independently evaluated all scans. Tumor responses were evaluated using the Response Evaluation Criteria in Solid Tumor (RECIST) 1.0. CA19-9 levels were measured at baseline and at each cycle. Clinical assessments of patient safety were performed by the investigators to identify any treatment- or non-treatment-related adverse events. Weekly laboratory testing was performed at our institutional central laboratory. Patients were followed until death.

### End points

The primary endpoints of this trial were overall survival (OS) and progression-free survival (PFS). Secondary endpoints included objective response rate (RR) and safety of chemotherapeutic agents. OS was calculated from the 1^st^ day of chemotherapy administration to the date of death or last follow-up. PFS was calculated from the first day of chemotherapy administration to the date of the first evidence of disease progression or last follow-up. Response was defined as partial response (PR), stable disease (SD), or progressive disease (PD) according to the Response Evaluation Criteria in Solid Tumors (RECIST). In our study, disease control rate (DCR) was defined as complete response (CR) plus partial response (PR), and prolonged stable disease. Response rate (RR) =CR+PR.

### Statistical analysis

The Kaplan-Meier method was used to estimate OS and PFS. Log rank test was performed for multiple comparisons between survival curves. Quantitative variables were compared by the Mann-Whitney U-test, and Fisher's exact test was used to assess qualitative variables. Log regression analysis was employed to analyze the effects of clinical factors on patients' drug susceptibility. All analyses were performed with the SPSS 17.0 software (SPSS, Chicago, IL, USA). *P*<0.05 was considered statistically significant.

## SUPPLEMENTARY TABLES


