# Supplementary material for: Combined gemcitabine and S-1 chemotherapy for treating unresectable hilar cholangiocarcinoma: a randomized open-label clinical trial
Source: Oncotarget. 2016 Apr 5;7(18):26888–97. doi: 10.18632/oncotarget.8590 (PMC5042023; doi:10.18632/oncotarget.8590)
Supplement: Supplementary file 1 [file oncotarget-07-26888-s001.pdf]

SUPPLEMENTARY TABLES

Supplementary Table S1: Baseline characteristics of patients

Characteristic	Total(n=75) No (%)	GEM(n=25) No (%)	S-1(n=25) No (%)	GEM-S-1(n=25) No (%)	P
Gender					
Male	54(72.0%)	16(64.0%)	19(76.0%)	19(76.0%)	0.551
Female	21(28.0%)	9(36.0%)	6(24.0%)	6(24.0%)	
Mean age (years)	56.3±7.83	55.1±8.5	56.8±7.9	57.0±7.2	0.497
Median CA19-9 (U/ml)	571 (466, 675)	584(424, 714)	620(492.5, 698)	514(436.5,622)	0.126
Median CEA (ng/ml)	2.50 (1.80, 3.10)	2.6(1.75, 3.05)	2.2(1.7, 3.05)	2.7(1.95, 3.25)	0.400
Bithmuth-Corlette classification					
IIIa	24 (32%)	8(32%)	8(32%)	8(32%)	1.000
IIIb	24 (32%)	8(32%)	8(32%)	8(32%)	
IV	27 (36%)	9(36%)	9(36%)	9(36%)	
UICC stage					
III	22(29.3%)	6(24%)	7(28%)	9(36%)	0.875
IVa	28(37.3%)	11(44%)	9(36%)	8(32%)	
IVb	25(33.3%)	8(32%)	9(36%)	8(32%)	
Metastasis					
Liver metastases	25(33.3%)	8(32%)	9(36%)	8(32%)	0.942
No metastases	50(66.7%)	17(68%)	16(64%)	17(68%)	
Biliary drainage					
PTCD	32(42.7%)	12(48%)	12(48%)	8(32%)	0.418
ERCP	43(57.3%)	13(52%)	13(52%)	17(68%)	

Abbreviations: GEM, gemcitabine; GEM-S-1, gemcitabine plus S-1; CA19-9, carbohydrate antigen 19-9; CEA, carcinoembryonic antigen; PTCD, percutaneous transhepatic catheter drainage; ERCP, endoscopic retrograde cholangiopancreatography.

Supplementary Table S2: Univariate and multivariate analysis of prognostic factors in patients with hilar cholangiocarcinoma

Factor	Univariate analysis			Multivariate analysis	
	No. of patients	One-year survival rate (%)	P	OR (95%CI)	P
Gender					
Male	54	22.2%	0.321		
Female	21	33.3%			
Age, y					
≧60	29	27.6%	0.722		
<60	46	23.9%			
CA19-9					
<500	26	69.2%	<0.001	55.556 (6.211-500)	<0.001
≧500	49	2.0%			
UICC Stage					
III	22	63.6%	<0.001	3.831 (1.060-13.889)	0.040
IVa	28	14.3%			
IVb	25	4.0%			
Bithmuth-Corlette classification					
IIIa	24	25.0%	0.996		
IIIb	24	25.0%			
IV	27	25.9%			
Metastasis					
Liver metastases	25	4.0%	0.003		
Without liver metastases	50	36.0%			
Chemotherapy regimens					
GEM	25	28.0%	0.032		
S-1	25	8.0%			
GEM-S-1	25	40.0%			

Abbreviations: CA19-9, carbohydrate antigen 19-9; UICC, International Union for Cancer Control; PTCD, percutaneous transhepatic catheter drainage; ERCP, endoscopic retrograde cholangiopancreatography; GEM, gemcitabine; GEM-S-1, gemcitabine plus S-1; OR, odds ratio; CI, confidence intervals.
